# Comparative Effects of Pterostilbene and Its Parent Compound Resveratrol on Oxidative Stress and Inflammation in Steatohepatitis Induced by High-Fat High-Fructose Feeding

**DOI:** 10.3390/antiox9111042

**Published:** 2020-10-24

**Authors:** Saioa Gómez-Zorita, Maitane González-Arceo, Jenifer Trepiana, Leixuri Aguirre, Ana B Crujeiras, Esperanza Irles, Nerea Segues, Luis Bujanda, María Puy Portillo

**Affiliations:** 1 Nutrition and Obesity group, Department of Nutrition and Food Science, Faculty of Pharmacy, University of the Basque Country (UPV/EHU), Lucio Lascaray Research Centre, 01006 Vitoria-Gasteiz, Spain; saioa.gomez@ehu.eus (S.G.-Z.); maitane.gonzalez@ehu.eus (M.G.-A.); leixuri.aguirre@ehu.eus (L.A.); irlesvidal.esperanza@gmail.com (E.I.); mariapuy.portillo@ehu.eus (M.P.P.); 2 BIOARABA Institute of Health, 01009 Vitoria-Gasteiz, Spain; 3 CIBERobn Physiopathology of Obesity and Nutrition, Institute of Health Carlos III (ISCIII), 01006 Vitoria-Gasteiz, Spain; anabelencrujeiras@hotmail.com; 4 Epigenomics in Endocrinology and Nutrition Group, Instituto de Investigación Sanitaria (IDIS), Complejo Hospitalario Universitario de Santiago (CHUS/SERGAS), 15704 Santiago de Compostela, Spain; 5 Department of Gastroenterology, University of the Basque Country (UPV/EHU), Donostia Hospital, 00685 San Sebastián, Spain; nereamiren.seguesmerino@osakidetza.eus (N.S.); luis.bujanda@osakidetza.net (L.B.); 6 BIODONOSTIA Institute of Health, 00685 San Sebastián, Spain; 7 CIBERehd Hepatic and Digestive Pathologies, Institute of Health Carlos III (ISCIII), 01006 Vitoria-Gasteiz, Spain

**Keywords:** pterostilbene, resveratrol, (poly)phenols, liver steatosis, liver steatohepatitis, hepatocarcinoma, oxidative stress, inflammation, rat

## Abstract

Different studies have revealed that oxidative stress and inflammation are crucial in NAFLD (Non-alcoholic fatty liver disease). The aim of this study is to analyze whether pterostilbene and resveratrol are able to either avoid or delay the progression of non-alcoholic liver steatosis towards steatohepatitis. This has been performed by examining their effects on oxidative stress, inflammation, fibrosis and pre-carcinogenic stages. Rats were distributed into five experimental groups and were fed with either a standard diet or a high-fat high-fructose diet, supplemented or not with pterostilbene (15 or 30 mg/kg/d) or resveratrol (30 mg/kg/d), for 8 weeks. Liver histological analysis was carried out by haematoxylin–eosin staining. Serum and hepatic oxidative stress-related parameters were assessed using spectrophotometry, and the expression of genes related to inflammation, fibrosis and cancer by qRT-PCR. The dietary model used in this study led to the development of steatohepatitis, where rats displayed oxidative stress, inflammation and ballooning, although not fibrosis. It also modified the expression of hepatocarcinoma-related genes. The results show, for the first time, that pterostilbene was able to partially prevent these alterations, with the exception of changes in hepatocarcinoma-related genes, mainly at 30 mg/kg/d. Pterostilbene was more effective than its parent compound resveratrol, probably due to its high bioavailability and higher anti-oxidant and anti-inflammatory activities, attributable to its different chemical structure.

## 1. Introduction

Non-alcoholic fatty liver disease (NAFLD) has become a global health problem affecting around 17–33% of the population worldwide [1]. It is characterized by excessive fat accumulation in the liver, not related to alcohol consumption or any other liver diseases [2]. A pathological broad spectrum of conditions is associated with NAFLD, ranging from simple steatosis to steatohepatitis (NASH), which can lead to cirrhosis and hepatocarcinoma.

Different animal studies and clinical trials have revealed that oxidative stress and inflammation are crucial in the pathophysiology of NAFLD. It has been observed that oxidative stress can increase inflammation, which in turn can boost the oxidative stress, thus resulting in a vicious cycle that promotes the progression of fatty liver to steatohepatitis [3,4].

Oxidative stress can be defined as a disturbance in the balance between the intrinsic antioxidant defence system and the formation of endogenous reactive oxygen species (ROS), which induce oxidative damage in biological molecules, such as lipids and proteins [5]. ROS include different molecules, like hydroxyl radicals, superoxide anion radicals and lipid peroxides, among others, that can be neutralized by enzymatic or non-enzymatic antioxidants. Regarding enzymatic defences, superoxide dismutase (SOD) catalyzes the dismutation of superoxide anion (O_2_^−^) into molecular oxygen (O_2_) and hydrogen peroxide (H_2_O_2_). This H_2_O_2_ is less reactive than ROS. Nevertheless, not only can it be toxic for the cells, and but it can also be transformed into hydroxyl radicals. H_2_O_2_ is degraded into water and O_2_ by catalase, mainly in the peroxisomes, and by glutathione peroxidase (GPx) in the mitochondria and cytosol. GPx also converts lipid peroxides into their corresponding alcohols [6].

A long-term oxidative stress can induce chronic inflammation through the NF-ĸB pathway, p53 or erythroid 2-related factor 2 (Nrf2), which could regulate the expression of inflammatory cytokines [7]. On the other hand, sustained inflammation could also induce oxidative stress. The phagocytic and non-phagocytic cells are able to produce large amounts of ROS in response to the pro-inflammatory cytokines [8].

With regard to the relationship between these two processes and hepatocarcinoma, it has been observed that ROS and pro-inflammatory molecules can act as carcinogens, causing DNA damage and mutations, as well as promote cell proliferation [9,10]. This outcome can be observed through the induction of genes associated with the initial phase of liver tumorigenesis, before the hepatocarcinoma becomes evident [11].

Since the options to manage NAFLD are limited, the scientific community is looking for new preventive and therapeutic agents. Oxidative stress being one of the causes of the progression of NAFLD, antioxidants like polyphenols are one of the most studied groups of molecules. Resveratrol is a polyphenol that has clear beneficial effects on liver steatosis in animal models [12,13]. The reported results in humans are controversial, although positive effects have been described in several studies [14,15]. However, the effects of resveratrol can be limited due to its low bioavailability [16]. For that reason, structural analogues of resveratrol with higher viability are being studied. Pterostilbene is a methoxylated derivative of resveratrol, in which the substitution of two hydroxyl groups with two methoxy groups improves its oral bioavailability [17]. As far as the prevention and treatment of NAFLD go, the effects of this phenolic compound have been much less studied than those of resveratrol.

In the cohort of rats used in the present study, we previously observed that high-fat, high-fructose feeding induced microvesicular steatosis, which was the expected type of steatosis considering that the experimental period length was 8 weeks [18,19]. This lipid alteration was partially prevented by the administration of both pterostilbene and resveratrol. This effect was seen in the histological analysis—steatosis was reduced by 49% in rats treated with pterostilbene at a dose of 15 mg/kg/d and by 87% in the group treated with pterostilbene or resveratrol at a dose of 30 mg/kg/d. The values of transaminases were also significantly reduced—in the case of alanine aminotransferase (ALT), the values were reduced by 45%, 55% and 48% in PT15, PT30 and RSV30 groups, respectively. Similarly, aspartate aminotransferase (AST) values were reduced by 39% in the PT15 rat group, by 38% in the PT30 group and by 29% in the RSV30 group.

After checking the anti-steatotic effect of both phenolic compounds, we were interested in analyzing whether they were able to avoid or delay the progression of non-alcoholic liver steatosis towards steatohepatitis, because very little is known concerning the potential benefits of resveratrol on this pathology. Furthermore, no information has been reported so far in relation to pterostilbene. For this purpose, we assessed the effects on oxidative stress of these compounds, as well as the liver inflammation of rats fed with a high-fat high-fructose diet. Moreover, their effects on pre-carcinogenic stages were also assessed.

## 2. Material and Methods

### 2.1. Animals, Diets and Experimental Design

Fifty male Wistar Rats (6-week-old; 140–150 g), purchased from Envigo (Barcelona, Spain), were housed in pairs in polycarbonate cages and placed in an air-conditioned room (22 ± 2 °C) in a 12 h light–dark cycle. After a 6-day adaptation period, rats were randomly distributed into 5 experimental groups (*n* = 10) and fed with experimental diets for 8 weeks. The control group (CC) was fed a commercial standard diet (AIN-93G, OpenSource Diets, Gentofte, Denmark, D10012G) and rats in the high-fat high-fructose group (HFHF) a diet containing 40% of lipids and 22% of fructose (OpenSource Diets, Gentofte, Denmark, D09100301). The other three groups received the same high-fat high-fructose diet, which was enhanced with the phenolic compounds. Rats in the PT15 and PT30 groups received pterostilbene at doses of 15 mg/kg body weight/d or 30 mg/kg body weight/d, respectively. In the RSV30 group, rats received resveratrol at a dose of 30 mg/kg body weight/d. With regard to the dose selection, in the case of resveratrol, in a previous study we analyzed the effects of different doses (6, 30 and 60 mg/kg) on obesity, and we observed that the most effective one was 30 mg/kg/d [20]. In further studies, we used this dose and we observed that it was effective in reducing liver steatosis [21,22]. Moreover, by revising the literature, we observed that this dose was in the range of the doses more commonly used by other authors [12]. In the case of pterostilbene, we selected the dose of 30 mg/kg/d in order to compare the effects of resveratrol and pterostilbene under the same conditions. It is well known that pterostilbene shows higher bioavailability than resveratrol [23]. This could lead to the effects of pterostilbene at 30 mg/kg/d being greater than those of resveratrol at the same dose. Considering that, depending on the dose range, resveratrol can reach a plateau in its effectiveness, or even that lower doses can induce greater effects than higher doses, we believe that the use of 15 mg/kg/d was interesting. Pterostilbene was kindly supplied by Chromadex (Irvine, CA, USA) and resveratrol by Monteloeder (Elche, Alicante, Spain). All animals had free access to food and water. Food intake and body weight were measured on a daily basis.

At the end of the experimental period (8 weeks), and after 12 h of fasting, animals were euthanized under anaesthesia (chloral hydrate) by cardiac exsanguination. Thee liver was dissected and weighed. Serum was obtained from blood samples after centrifugation (1000× *g*, 10 min, 4 °C). All samples were stored at −80 °C until analysis. The experiment was performed in agreement with the Ethical Committee of the University of the Basque Country (document reference M20_2015_245 CUEID), following the European regulations (European Convention-Strasburg 1986, Directive 2003/65/EC and Recommendation 2007/526/EC).

### 2.2. Histopathological Evaluation of NAFLD

To perform a histological study by light microscopy right after sacrifice, liver samples were fixed in 10% buffered formalin and subsequently embedded in paraffin. Liver sections were stained with both haematoxylin and eosin along with Masson’s trichrome using standard techniques. The treatment group each animal belonged to was deliberately concealed during the analysis of the sections.

The evaluated features were steatosis, lobular inflammation, ballooning degeneration and fibrosis. Lobular inflammation was graded based on the number of inflammatory foci per 200 × field—0 when there were no inflammatory foci, 1 when there was 1 to 2 inflammatory foci per 200 × field, and 2 when 2 to 4 inflammatory foci per 200 × were found. Ballooning was evaluated based on numbers of hepatocytes showing this lesion—0 when there was no ballooning, 1 when few hepatocytes showed ballooning, and 2 when many cells showed ballooning degeneration. Finally, the NAFLD Activity Score (NAS) was calculated using data from steatosis, lobular inflammation, ballooning and fibrosis scores.

### 2.3. Serum Uric Acid Concentration

Uric acid was determined in serum samples by using a commercial kit (SpinReact, Girona, Spain).

### 2.4. Parameters Related to Oxidative Stress in Liver

#### 2.4.1. Lipid Peroxidation Measurement

Lipid peroxidation in rat liver lysates was determined by a commercial TBARS assay kit (Cayman Chemical¸ Ann Arbor, MI, USA). The method is based on the determination of thiobarbituric acid reactive substances (TBARS) as a marker for lipid peroxidation. The method uses the reaction of malondialdehyde (MDA) and thiobarbituric acid (TBA) in an acid medium. MDA-TBA adduct can be measured in an Infinite 200Pro plate reader (Tecan, Männedorf, Zürich, Switzerland). Results were expressed as µg MDA/mg of tissue.

#### 2.4.2. Total Antioxidant Capacity Determination

The total antioxidant capacity in rat liver homogenates was determined by using the commercial kit OxiSelect Oxygen Radical Antioxidant Capacity (ORAC) activity assay (Cell Biolabs, San Diego, CA, USA). The ORAC assay was carried out using fluorescein as a fluorescence probe. After that, the free radical initiator AAPH (2,2-azobis(2-amidinopropane) dihydrochloride) utilized to produce peroxyl radicals was added to the sample and the fluorescence was reordered in an Infinite 200Pro plate reader (Tecan, Männedorf, Zürich, Switzerland). Trolox solution was used for building the calibration curve. Results were calculated based upon differences in areas under the fluorescence decay curve among blank, samples and standards. Final ORAC values were expressed as µM Trolox equivalents/mg protein.

#### 2.4.3. Determination of Reduced Glutathione

Reduced glutathione (rGSH) concentrations were measured using the glutathione colorimetric assay kit (Biovision, Milpitas, CA, USA). The assay is based on the glutathione recycling system in the presence of GSH and the DTNB fluorophore. The reduction of DTNB produces a stable fluorescent product which can be detected in an Infinite 200Pro plate reader (Tecan, Männedorf, Zürich, Switzerland). Results were expressed as ng/mg of tissue.

#### 2.4.4. Superoxide Dismutase Activity

Total superoxide dismutase (SOD; EC 1.15.1.1) activity in rat liver lysate was measured by the SOD activity assay kit (Biovision, Milpitas, CA, USA). This method uses the xanthine–xanthine oxidase system to generate superoxide anions. The superoxide anion reduces WST-1, which is converted into WST-1 formazan. The absorbance of this reduced form is recorded on an Infinite 200Pro plate reader (Tecan, Männedorf, Zürich, Switzerland). In the presence of SOD, O_2_^−^ undergoes a dismutation into O_2_ and H_2_O_2_, thus decreasing the WST-1 formazan formation. As such, this competing assay yields to the indirect measurement of SOD activity. The SOD activity (% inhibition rate) was calculated according to the manufacturer’s instruction.

#### 2.4.5. Catalase

Catalase (CAT; EC 1.11.1.6) activity was measured according to Aebi (1984) [24], by observing spectrophotometrically the H_2_O_2_ disappearance at 240 nm. The reaction took place in a final volume of 250 µL containing 90 mM potassium phosphate buffer (pH 6.8) and started with the addition of H_2_O_2_ (37.5 mM final concentration). Catalase activity was expressed as nmol/min/µg of protein.

#### 2.4.6. Glutathione Peroxidase

Glutathione peroxidase (GPx; EC 1.11.1.9) activity in rat liver lysate was evaluated by measuring the H_2_O_2_ scavenging capacity using the GPx assay kit (Biovision, Milpitas, CA, USA) according to the manufacturer’s instructions. GSSG, produced upon reduction of H_2_O_2_ by GPx, was recycled in its reduced state by both glutathione reductase and reduced nicotinamide adenine dinucleotide phosphate (NADPH). The decrease in absorbance of NADPH was monitored in an Infinite 200Pro plate reader (Tecan, Männedorf, Zürich, Switzerland). Results were expressed as GPx U/mg of protein.

#### 2.4.7. Determination of Total Proteins

Total protein was spectrophotometrically quantified in lysates and homogenates at 595 nm by Bradford assay [25], using bovine serum albumin as standard.

#### 2.4.8. Extraction, Reverse Transcription and Quantification of RNA by Real-Time Polymerase Chain Reaction (PCR)

Total RNA was isolated from 100 mg of liver using Trizol (Invitrogen, Carlsbad, CA, USA), according to the manufacturer’s instructions. After DNase treatment (Ambion, Foster City, CA, USA), the integrity of the RNA was verified and quantified using an RNA 6000 Nano Assay (Thermo Scientific, Wilmington, DE, USA). RNA samples were then treated with a DNA-free kit (Ambion, CA, USA). Measurements of 1.5 μg of total RNA from each sample were reverse-transcribed into complementary DNA (cDNA) using iScript cDNA Synthesis Kit (Bio-Rad, Hercules, CA, USA).

Actin alpha 2 smooth muscle (*Acta2*), ARM protein lost in epithelial cancers on chromosome X 3 (*Alex3)*, BCL2 associated X protein (*Bax)*, macrophage mannose receptor 1 (*Cd206*), collagen type I alpha 1 chain (*Col1α1*), C-reactive protein (*Crp)*, adhesion G protein-coupled receptor E1 (*F4/80)*, fibroblast growth factor 21 (*Fgf21*), glutathione S-transferase mu 2 (*Gstm2)*, interleuquin 1 beta (*Il1β)*, myeloid differentiation factor 88 (*MyD88)*, NADPH oxidase 4 (*Nox4)*, cytochrome B-245 alpha chain (*P22phox)*, sirtuin 1 (*Sirt1*), survivin *(Birc5)*, telomerase reverse transcriptase *(Tert)*, transforming growth factor beta 1 (*Tgf β1*), TIMP metallopeptidase inhibitor 1 (*Timp1*), toll-like receptor 2 and 4 (*Tlr-2, Tlr-4*) and tumor protein P53 (*Tp53)* genes were quantified, as well as β-Actin, which served as the reference gene in posterior normalization. A 4.75 μL aliquot of each diluted cDNA sample was used for Real-Time PCR amplification in a 12.5 μL reaction volume. The cDNA samples were amplified in an iCycler-MyiQ Real-Time PCR Detection System (Bio-Rad, Hercules, CA, USA), in the presence of SYBR Green Master Mix (Applied Biosystems, Foster City, CA, USA) and of the sense and antisense primers (300 nM each). The sequences are described in Table 1. The PCR parameters were as follows: initial 2 min at 50 °C, denaturation at 95 °C for 10 min followed by 40 cycles of denaturation at 95 °C for 15 s, annealing at 60 °C for 30 s and extension at 60 °C for 30 s. For *Il1β* and *Tgf β1*, the annealing temperature was 59 °C. Genes *Bcl2*, *CD206*, *Col1α1, Crp, MyD88, Nox4, Tgfβ1* and *Timp1* were amplified in 45 cycles.

Monocyte Chemoattractant Protein 1 (*Mcp1*) and tumor necrosis factor α (*Tnfα*) were amplified using TaqMan probes (Table 1). 18S mRNA levels were similarly measured and served as the reference gene. In total, 4.5 μL of each diluted cDNA sample was added to the PCR reagent mixture, which consisted of TaqMan Fast Advanced Master Mix (Applied Biosystems, Vilna, Lithuania) and TaqMan Gene Expression Assay Mix (Applied Biosystems, Foster City, Ca, USA) containing specific primers and probes (*18S*: Rn03928990; *Mcp1*: Rn00580555; *Tnfα*: Rn01525859). The PCR parameters were as follows: initial 2 min at 50 °C, denaturation at 95 °C for 2 min followed by 40 cycles of denaturation at 95 °C for 3 s and combined annealing and extension at 60 °C for 30 s.

In all cases, the results were expressed as fold changes of the threshold cycle (Ct) value relative to controls using the 2^−ΔΔCt^ method [26].

#### 2.4.9. Statistical Analysis

The results are presented as means ± standard error of the means. Statistical analysis was performed using SPSS 25.0 (SPSS Inc. Chicago, IL, USA). The normal distribution of the data was confirmed by Shapiro–Wilks test. Data were analyzed by one-way ANOVA followed by Tukey post-hoc test. Statistical significance was established at the *p* < 0.05 level.

## 3. Results

### 3.1. Histopathological Assessment

**Figure 1:**
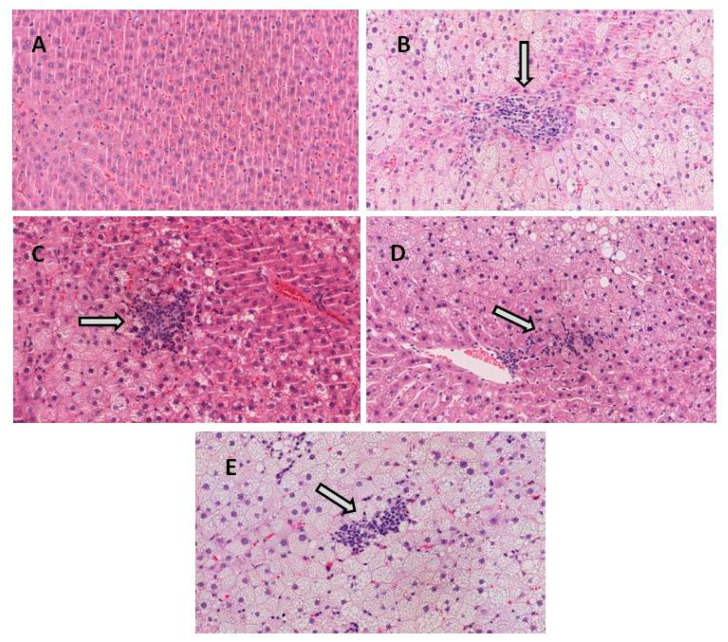
Representative H&E stained histological liver samples (magnification ×20). (**A**) healthy liver from CC group, (**B**) liver from HFHF group showing moderate inflammation and ballooning degeneration, (**C**) liver from PT15 group showing moderate inflammation and ballooning degeneration, (**D**) liver from PT30 group showing mild inflammation and ballooning degeneration and (**E**) liver from RSV30 group showing mild–moderate inflammation and ballooning degeneration. White arrows indicate inflammation.

When the histological characteristics of the livers were evaluated by microscopy (*n* = 10 in all groups of rats), the control (CC) group expectedly showed no lobular inflammation, ballooning degeneration or fibrosis. By contrast, in the high-fat high-fructose (HFHF) group, 50% of the animals showed mild inflammation whilst the other 50% exhibited moderate inflammation. Additionally, all of them presented prominent ballooning (Table 2 and Figure 1).

The treatment with pterostilbene, at a dose of 15 mg/kg body weight/d, reduced lobular inflammation (20% of animals showed moderate inflammation, whereas 80% of them displayed mild inflammation). Treatment with pterostilbene at a higher dose better reduced lobular inflammation. In the PT30 group, the phenolic compound avoided lobular inflammation in 20% of the animals, whereas 80% showed mild inflammation. In the case of resveratrol treatment, 30% of the animals exhibited moderate inflammation and 70% mild inflammation. Finally, phenolic compounds were not able to reduce ballooning (Table 2 and Figure 1). NAS score, which shows values in the range 0–8, was strongly increased in the HFHF group when compared to the CC group, and it was reduced in all groups that received the phenolic compounds when compared to the HFHF group (Table 2). Regarding fibrosis, none of the animals showed hepatic fibrosis, with the exception of one rat from the HFHF group that showed mild perisinusoidal fibrosis (Table 2).

### 3.2. Serum Uric Acid Concentration

High-fat, high-fructose feeding induced an increase in serum uric acid concentration (+ 31.2%), but this did not reach statistical significance (1.6 ± 0.2 in the control group vs. 2.1 ± 0.2 in HFHF group, expressed as mg/dL). No significant changes were observed in rats treated with the phenolic compounds when compared to the HFHF group (1.7 ± 0.1 in PT15 group, 2.1 ± 0.2 in PT30 group and 2.2 ± 0.2 in RSV30 group, expressed as mg/dL).

### 3.3. Hepatic Oxidative Stress Markers

**Figure 2:**
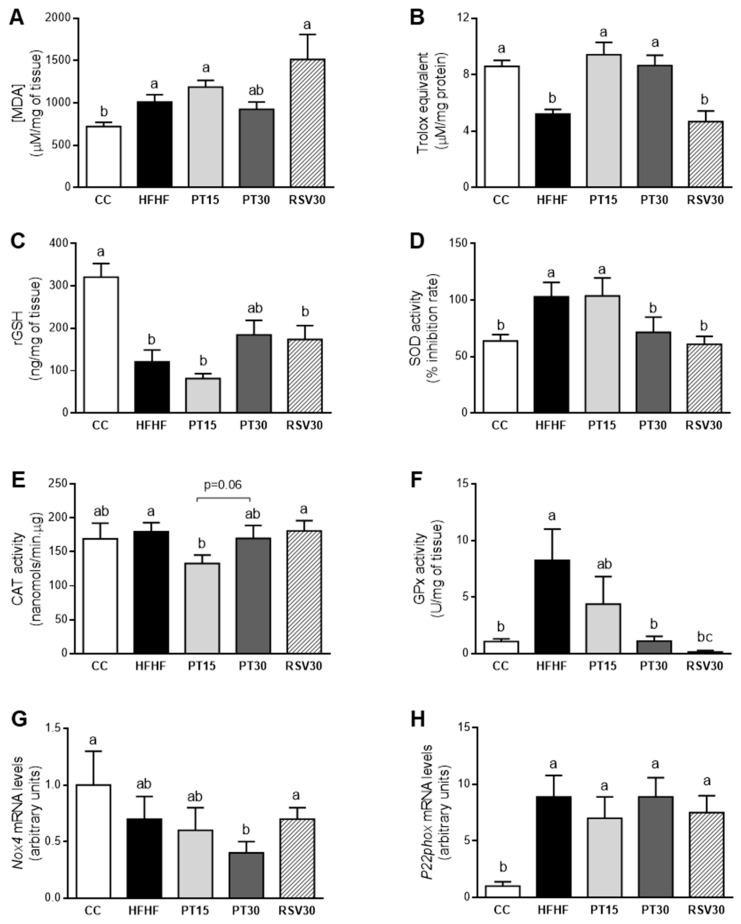
Lipid peroxidation (MDA content), ORAC, rGSH, activities of SOD, CAT and GPx, and gene expression of *Nox4* and *P22phox* in liver from the rats fed with a control diet (CC group), a high-fat high-fructose diet (HFHF group), a high-fat high-fructose diet supplemented with pterostilbene at a dose of 15 mg/kg body weight/d (PT15 group), a high-fat high-fructose diet supplemented with pterostilbene at a dose of 30 mg/kg body weight/d (PT30 group) or a high-fat high-fructose diet supplemented with resveratrol at a dose of 30 mg/kg body weight/d (RSV30 group) (**A**–**H**). Values are presented as mean ± SEM. Bars not sharing common letters a–c are significantly different (*p* < 0.05). MDA: malondialdehyde; ORAC: Oxygen Radical Antioxidant Capacity; rGSH: reduced glutathione; SOD: superoxide dismutase; CAT: catalase; GPx: glutathione peroxidase; *Nox4*: NADPH oxidase 4; *P22phox*: cytochrome B-245 alpha chain.

When oxidative stress was evaluated, rats fed with the HFHF diet showed a significant increase in lipid peroxidation (MDA; Figure 2A). Moreover, in these animals the antioxidant capacity (ORAC; Figure 2B) and the non-enzymatic antioxidant rGSH (Figure 2C) was decreased. However, the HFHF diet induced the activation of the antioxidant enzymes SOD (Figure 2D) and GPx (Figure 2F), whereas no changes were observed in CAT activity (Figure 2E).

With regard to the effects of the phenolic compounds, lipid peroxidation in rats from the PT30 group showed intermediate values among those from the CC and HFHF groups (Figure 2A). In addition, both pterostilbene doses were able to restore the antioxidant capacity; in fact, rats from both PT groups displayed similar values to those found in the control group (Figure 2B). Regarding the non-enzymatic protection, the high dose of pterostilbene partially avoided the rGSH reduction induced by the high-fat high-fructose diet, although the change did not reach statistical significance (+44%, *p* = 0.078 vs. HFHF group; Figure 2C). In the case of antioxidant enzyme activities, the high dose of pterostilbene completely restored the control values of SOD and GPx (Figure 2D,F). The low dose of this phenolic compound significantly reduced CAT antioxidant activity, when compared to the HFHF group (–26%; Figure 2E). In resveratrol-treated rats, only SOD and GPx activities were significantly modified when compared to the HFHF group; the antioxidant activity of these enzymes achieved a total restoration (Figure 2D,F).

Finally, high-fat high-fructose feeding significantly increased *P22phox* mRNA levels, whereas no changes were observed in *Nox4* gene expression. The increase induced in *P22phox* by HFHF feeding was not prevented by the phenolic compounds. What is more, the high dose of pterostilbene significantly reduced *Nox4* gene expression below the level of the control group (Figure 2G,H).

### 3.4. Gene Expression of Inflammation-Related Markers in the Liver

**Figure 3:**
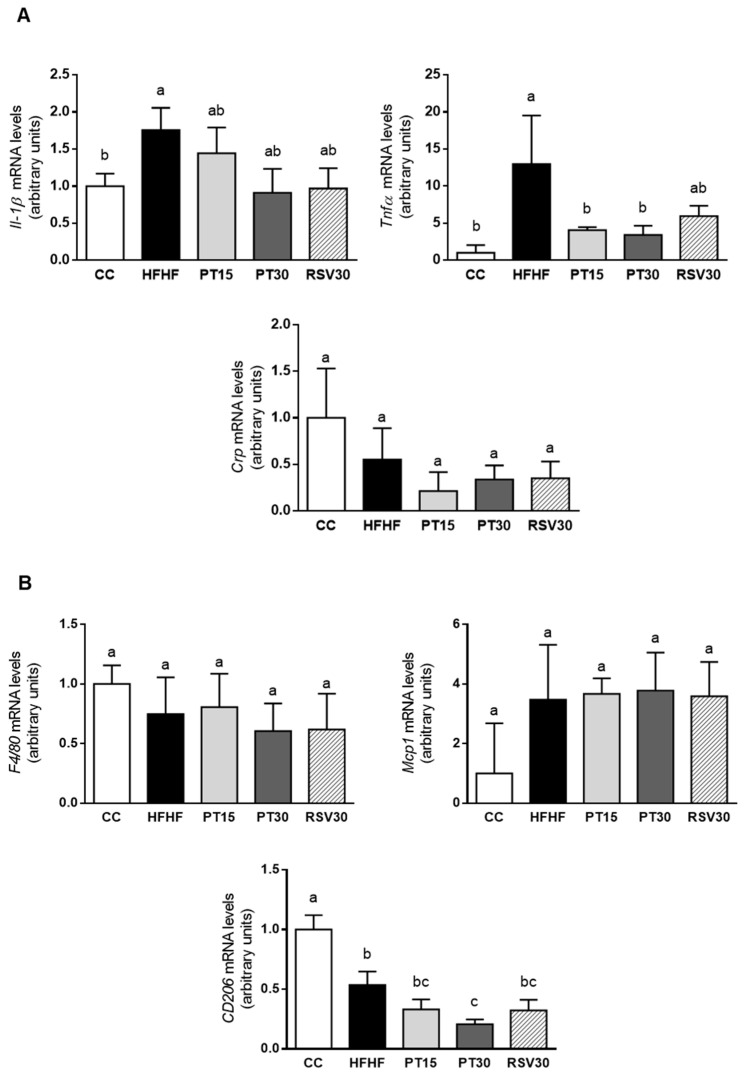

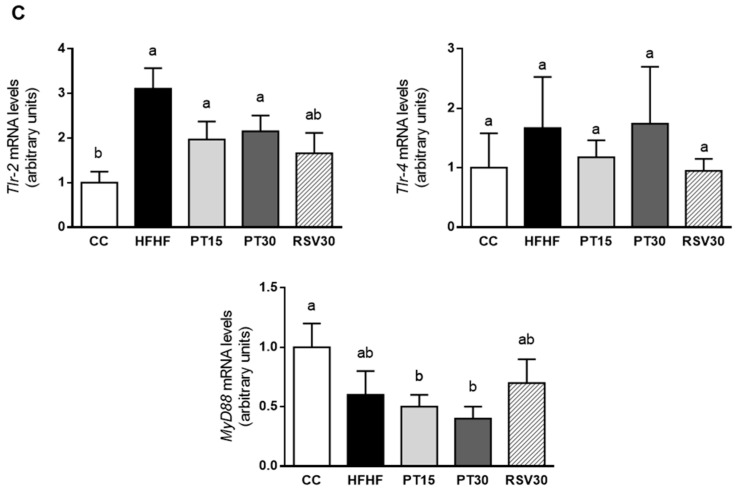
*Il-1β*, *Tnfα* and *Crp* mRNA levels (**A**), *F4/80*, *Mcp1* and *Cd206* mRNA levels (**B**) and *Tlr-2*, *Tlr-4* and *MyD88* mRNA levels (**C**) in the livers from the rats fed with a control diet (CC group), a high-fat high-fructose diet (HFHF group), a high-fat high-fructose diet supplemented with pterostilbene at a dose of 15 mg/kg body weight/d (PT15 group), a high-fat high-fructose diet supplemented with pterostilbene at a dose of 30 mg/kg body weight/d (PT30 group) or a high-fat high-fructose diet supplemented with resveratrol at a dose of 30 mg/kg body weight/d (RSV30 group). Values are presented as mean ± SEM. Bars not sharing common letters are significantly different (*p* < 0.05). *Il-1b*: interleukin 1b; *Tnfα*: tumor necrosis factor α; *Crp*: C-reactive protein; *Mcp1*: monocyte chemoattractant protein 1; *Cd206*: mannose receptor C; *Tlr-2* and *Tlr-4*: toll-like receptor 2 and 4; *MyD88*: myeloid differentiation factor 88 *MyD88*.

The mRNA levels of the inflammatory cytokines in the liver are shown in Figure 3. Animals fed the high-fat high-fructose diet displayed higher expression levels of *Il-1ß* and *Tnf-α*. In the case of *Il-1ß*, animals treated with either pterostilbene or resveratrol showed intermediate values between the CC and the HFHF groups. However, *Tnf-α* expression levels decreased significantly in the two groups treated with pterostilbene. No differences were observed in *Crp* mRNA levels among the experimental groups (Figure 3A).

Regarding macrophage infiltration, *MCP*, a marker of M1 macrophages, and *CD206*, a marker of M2 macrophages, were measured. In the HFHF group, *Mcp1* gene expression increased by 250%, although no statistical difference was achieved due to the high value of the standard error of the mean. Furthermore, *CD206* gene expression was significantly decreased when compared to the control group. The phenolic compounds did not avoid these effects, with the exception of the expression of *CD206* in the PT30 group, which was significantly reduced when compared to the HFHF group. *F4/80*, a protein involved in the generation of antigen-specific efferent regulatory T (T reg) cells, was also measured, and no significant differences were observed among the experimental groups (Figure 3B).

With regard to TLRs, *Tlr-2* expression was increased in the HFHF group when compared to the CC group. This effect was partially prevented by the treatment with the phenolic compounds (−37%, −31% and −47% in the PT15, PT30 and RSV30 groups, respectively). No differences among groups were observed in *Tlr-4* gene expression. Lastly, the expression of *MyD88*, a gene that codifies for a protein used by the Toll-like receptor aimed at activating the transcription factor NF-κB, was not significantly modified by the steatotic diet. The PT15 and PT30 groups showed lower values than the CC group did (Figure 3C).

### 3.5. Gene Expression of Fibrogenic Markers in the Liver

**Figure 4:**
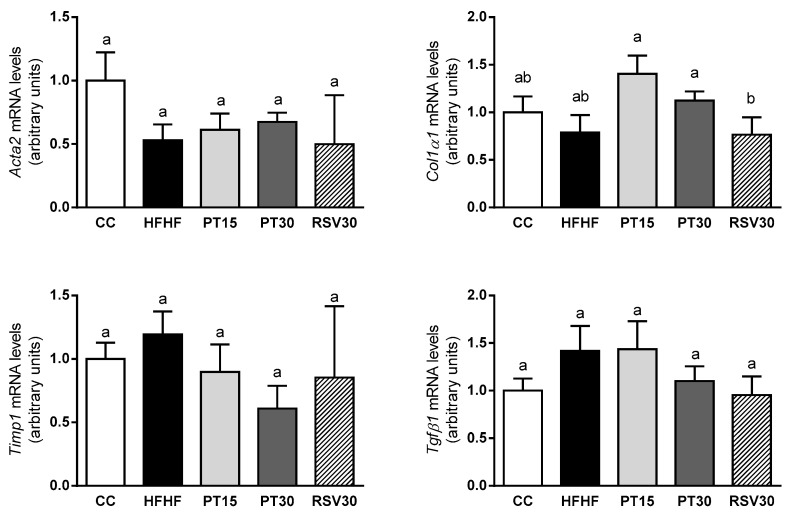
*Acta2*, *Col1a1*, *Timp1* and *Tgfβ1* mRNA levels in the livers from the rats fed with a control diet (CC group), a high-fat high-fructose diet (HFHF group), a high-fat high-fructose diet supplemented with pterostilbene at a dose of 15 mg/kg body weight/d (PT15 group), a high-fat high-fructose diet supplemented with pterostilbene at a dose of 30 mg/kg body weight/d (PT30 group) or a high-fat high-fructose diet supplemented with resveratrol at a dose of 30 mg/kg body weight/d (RSV30 group). Values are presented as mean ± SEM. Bars not sharing common letters a,b are significantly different (*p* < 0.05). *Acta2*: α-smooth muscle actin; *Col1a1*: collagen 1; *Timp*: tissue inhibitor of matrix metalloproteases; *Tgfβ1*: transforming growth factor beta1.

No differences were observed in *Tgfβ1*, *Col1α1*, *Timp1* and *Acta2* gene expression among experimental groups (Figure 4).

### 3.6. Gene Expression of Hepatocarcinoma Markers in the Liver

**Figure 5:**
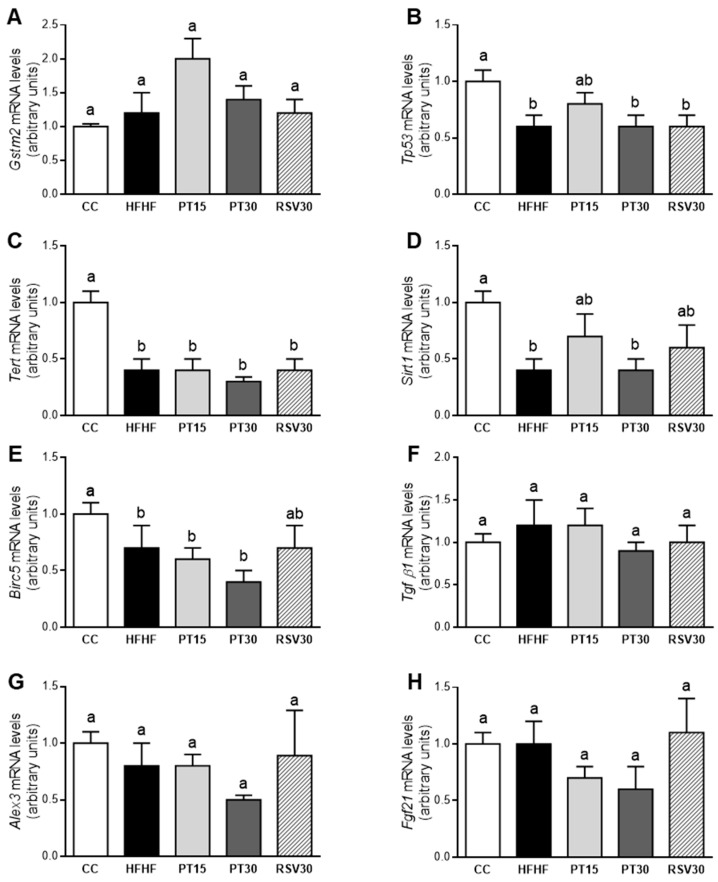
*Gstm2*, *Tp53*, *Tert*, *Sirt1*, *Birc5*, *Tgf β1*, *Alex3* and *Fgf21* mRNA levels in the livers from the rats fed with a control diet (CC group), a high-fat high-fructose diet (HFHF group), a high-fat high-fructose diet supplemented with pterostilbene at a dose of 15 mg/kg body weight/d (PT15 group), a high-fat high-fructose diet supplemented with pterostilbene at a dose of 30 mg/kg body weight/d (PT30 group) or a high-fat high-fructose diet supplemented with resveratrol at a dose of 30 mg/kg body weight/d (RSV30 group) (**A**–**H**). Values are presented as mean ± SEM. Bars not sharing common letters a,b are significantly different (*p* < 0.05). *Alex3*: ARM protein lost in epithelial cancers on chromosome X 3; *Birc5*: survivin; *Fgf21*: fibroblast growth factor 21; *Gstm2*: glutathione s-transferase mu2; *Sirt1*: sirtuin 1; *Tert*: telomerase reverse transcriptase; *Tgf β1*: transforming growth factor beta 1; *Tp53*: tumor protein P53.

High-fat high-fructose feeding decreased the gene expression of *Tp53*, *Tert*, *Sirt1* and *Birc5*. Pterostilbene, at a dose of 15 mg/kg body weight/d, partially prevented this decrease in *Tp53* and *Sirt1*. Surprisingly, the high dose of this phenolic compound induced no changes. Similar effects were observed in rats treated with resveratrol to those observed in the PT15 group, as well as a partial prevention of the decrease in *Birc5* (Figure 5).

## 4. Discussion

Non-alcoholic fatty liver is characterized by an excess of triglyceride accumulation in hepatocytes. The main features that differentiate this metabolic alteration from non-alcoholic steatohepatitis are hepatocyte injury and cell death exhibiting inflammation and very often fibrogenesis [27]. As was described in the Introduction section, we previously analyzed the steatosis induced by high-fat high fructose feeding and the preventive effects of pterostilbene and resveratrol in this precise cohort of rats. We observed a clear reduction in all the groups treated with the phenolic compounds. In the case of pterostilbene, this reduction was greater when the high dose of this compound was administered. At a dose of 30 mg/kg/d, the effectiveness of resveratrol and pterostilbene was similar. In the present manuscript, we focus our interest on steatosis progression to steatohepatitis, and thus the effects of the experimental treatments on inflammation and oxidative stress are presented.

Histological analysis shows that steatohepatitis was developed. Indeed, although fibrosis was not appreciated after 8 weeks, all rats in the HFHF group exhibited mild or moderate inflammation, in addition to steatosis. Moreover, all of them also displayed ballooning degeneration, which is a form of hepatocyte death generally considered as a form of apoptosis and a descriptor of steatohepatitis [28]. When the NAS score, which represents the sum of scores for steatosis, lobular inflammation and ballooning, was calculated, the mean value increased from 0 in the control group to 5.8 in HFHF group.

The functional alterations commonly found in NAFLD lead to the production of deleterious reactive oxygen species (ROS) and a decrease in antioxidant defences [29,30]. In this sense, ROS attack polyunsaturated fatty acids inducing lipid peroxidation in the cells, which leads to the subsequent MDA formation [31]. Therefore, we further investigated the oxidative status in this cohort of rats by measuring the hepatic MDA level, ORAC, rGSH, and the antioxidant enzyme activity. Our results show that the ability of the antioxidant defences to neutralize ROS diminished in rats under the high-fat high-fructose diet, since the antioxidant capacity and the non-enzymatic antioxidant GSH were decreased. Due to this oxidant/antioxidant imbalance, lipid peroxidation augmented, which suggests oxidative stress induction by the high-fat high-fructose diet in this experimental model, although ROS levels were not directly measured.

Regarding the endogenous enzymatic defences, the oxidative stress prompted by the high-fat high-fructose diet in this experimental model induced an increase in SOD enzyme activity, which could be interpreted as a compensatory mechanism aimed at minimizing the harmful effect of the ROS. Although controversial results have been reported with regard to changes in SOD activity observed in NAFLD models [32,33], our results are in good accordance with those shown in a rat model of insulin resistance induced by a high-fat diet, where the over-expression of mitochondrial SOD played a central role in counteracting the redox imbalance and protecting against the insulin resistance set off by the diet in rat skeletal muscle [34]. Moreover, in the present study, rats fed the high-fat high-fructose diet showed increased GPx enzyme activity. This result is in line with those reported by Perlemuter et al. (2005) in the livers of NAFLD patients. These authors observed increased Cu/Zn-SOD and GPx enzyme activity [35].

As far as uric acid is concerned, circulating levels are independently associated with NASH [36]. It has been suggested that uric acid might not be just an inert by-product of fructose metabolism, but instead a mediator of some of the hepatotoxic effects of fructose. Numerous studies have shown that uric acid can induce both cytosolic and mitochondrial oxidative stress by the activation of NADPH oxidase (Nox) [37,38]. In this metabolic pathway, Nox4 directly interacts with the P22phox protein to form the functional NADPH oxidase enzyme [39,40]. Moreover, uric acid can also cause oxidative stress directly [41].

In the present study, rats fed with the high-fat high-fructose diet showed an increase (+31.2%) in serum levels of this metabolite, but this reached no statistical significance. Regarding the Nox4-P22phox pathway, these rats showed unchanged levels of *Nox4* gene and a significant increase in *P22phox* gene expression. These results suggest that, under our experimental conditions, uric acid did not seem to be involved in the oxidative stress induced by high-fat high-fructose feeding.

It is known that pterostilbene plays a key role as a free radical scavenger, and that it protects against DNA damage induced by oxidative instability [42]. In the present study, pterostilbene at a dose of 30 mg/kg/d was able to partially avoid lipid peroxidation and to prevent the antioxidant capacity depletion induced by the high-fat high-fructose diet. This effect could be related to the partial prevention of the reduction in rGSH observed in the PT30 group when compared to the HFHF group. These results are consistent with those reported by other authors. On this topic, Kosuru and Sing (2017) observed restored GSH levels in fructose-fed diabetic rats under oral administration of pterostilbene, at a dose of 20 mg/kg/d [43]. With respect to antioxidant enzymes, the increase in both SOD and GPx activity prompted by the high-fat high-fructose diet was not observed in the PT30 group. This suggests that the compensatory mechanism achieved by the antioxidant enzymes in order to counteract the oxidative stress was no longer necessary. In general terms, these effects were not observed in the PT15 group, showing that under our experimental conditions, 15 mg/kg/d was not enough to prevent the oxidative stress produced by high-fat high-fructose feeding. With regard to resveratrol, it has been reported that it is less efficient than pterostilbene in reducing oxidative stress and DNA damage [44,45,46], as well as in inhibiting hepatic oxidative stress and lipid peroxidation [47]. This fact was confirmed in the present study.

Inflammation, observed by histology in HFHF group, was paralleled by gene expression, since the hepatic mRNA levels of *Il-1ß* and *Tnfα*, two cytokines that are relevant mediators in the development of NAFLD, were increased in HFHF rats. In addition, the gene expression of *Tlr-2* and *Mcp1* (a marker of the pro-inflammatory macrophages M1) was also increased, when in fact that of *CD206* (a marker of anti-inflammatory macrophages M2) was decreased. Taking into account that the activation of M1 macrophages, which is mediated by TLRs, leads to the production of inflammatory cytokines [48], the increase in *Tlr-2, Mcp1, Il-1ß* and *Tnfα* was a related feature.

Pterostilbene acted as an anti-inflammatory molecule because, at a dose of 15 mg/kg body weight/d, it reduced the number of rats that showed moderate inflammation. Moreover, after the treatment at 30 mg/kg body weight/d, no rats showed moderate inflammation and two rats displayed no inflammation at all. The increase induced by high-fat high-fructose feeding in hepatic *Il-1ß* gene expression was partially prevented by this molecule (–18% in PT15 group and –48% in PT30 group), and there was a significant reduction in *Tnfα* gene expression. Conversely, the anti-inflammatory effect of pterostilbene did not seem to be mediated by a reduction in M1 macrophage infiltration. With regard to TLRs, the partial prevention of the increase induced by the high-fat high-fructose feeding, and the reduction in MyD88, suggest the involvement of this pathway in the anti-inflammatory effect of this phenolic compound. Nonetheless, this issue requires further research. By contrast, ballooning degeneration was not prevented by pterostilbene.

Taking these results into account, and considering that pterostilbene also reduced steatosis, the values of the NAS score in the PT groups (3.8 ± 0.3 and 3.1 ± 0.3 for PT15 and PT30, respectively) were lower than those in the HFHF group (5.4 ± 0.4). Our data cannot be compared to other studies since no data concerning the effects of pterostilbene on steatohepatitis have been reported so far. Nevertheless, the effects observed in the present study are in line with the anti-inflammatory action of pterostilbene observed in several diseases, including neuroinflammation, dermatitis, pancreatitis, inflammatory bowel disease, atherosclerosis and obesity [49].

Resveratrol (30 mg/kg body weight/d) also showed an anti-inflammatory effect, although it is worth highlighting the fact that the extent of this effect was similar to that of pterostilbene at a dose of 15 mg/kg body weight/d. Thus, as observed in this cohort of rats, pterostilbene and resveratrol were equally efficient in preventing liver steatosis, although the former was more successful in preventing inflammation. The value of NAS in the RSV30 group was lower than that in the HFHF group (3.8 ± 0.3 vs. 5.4 ± 0.4), and similar to that observed in the PT15 group. As in the case of pterostilbene, the inflammation was not associated with macrophage infiltration. This fact has also been observed by other authors that have treated rodents with high-fat diets and resveratrol [50].

The absence of fibrosis development was evidenced by the histological analysis, as well as by the lack of change in the expression of genes related to this process, such as *Tgfβ1*, *Col1a1*, *Timp* and *Acta2*. This is in good accordance with the reported literature. In fact, a high-fat high-fructose diet does not always induce fibrosis [51,52].

A relevant issue to consider regarding the NAFLD pathophysiology is that this metabolic disorder contributes to the development of hepatocarcelullar carcinoma unrelated to viruses [53,54]. In this regard, although a complete study of carcinogenesis was out of the scope of the present work, because it was not induced in our experiment when the HFHF group was compared to the control group, we did measure several genes related to this process due to the relevance of having biomarkers for very early stages of alterations, even before the possible onset of the disease.

*Tp53* and *Sirt1* showed statistically significant lower expressions in the HFHF group. These genes being tumor suppressors, they can be regulated by oxidative stress, and they are involved in the protection against the initiation of tumorigenesis [55,56,57]. These results are concordant with the dysregulation of oxidative stress and inflammation induced in the HFHF group, and show that high-fat high-fructose feeding can represent a real risk in hepatocarcinoma development. It has been reported that *Birc5/survivin* decreases in the progression of NAFLD from hepatic steatosis to cirrhosis and hepatocarcinoma [58]. These data contrast with the fact that *Birc5/survivin* is up-regulated in malignant transformed cells because it is essential for cell division [59,60], and suggest that the control of cell cycle and of apoptosis may be quite different among different liver diseases [58]. Taking into account that *Birc5/survivin* is a specific inhibitor of caspases 3, 7 and 9, the explanation provided by several authors to understand the decrease in *Birc5/survivin* gene expression in the progression of steatohepatitis to hepatocarcinoma is that this change could be associated with an increased apoptosis for liver regeneration after hepatic injury [61]. On this topic, we were also able to observe a down-regulation of *Birc5/survivin* in the HFHF group compared to the control group, which also paralleled with the expression of *Tp53* and *Sirt1*. The treatments with pterostilbene or resveratrol did not significantly avoid the changes induced in those genes by high-fat high-fructose feeding.

In conclusion, the dietary model used in the present study leads to the development of hepatic steatohepatitis and shows oxidative stress, inflammation and ballooning, although it does not result in fibrosis. The present results show, for the first time, that pterostilbene was able to partially prevent these alterations, mainly at the dose of 30 mg/kg body weight/d. In view of these results, this phenolic compound could be considered as a useful tool to delay the evolution of steatosis to steatohepatitis. Clinical studies are needed to test if this positive action could also be found in human beings. Pterostilbene was more effective than its parent compound resveratrol, probably due to its high bioavailability and higher anti-oxidant and anti-inflammatory activities, which are attributed to its different chemical structure (the presence of two methoxy groups instead of two hidroxyl groups). To conclude, neither of the phenolic compounds are able to prevent the negative pattern of hepatocarcinoma-related genes induced by high-fat high-fructose feeding.

## Figures and Tables

**Table 1:** Primer sequences for quantitative Real-Time PCR amplification with SYBR Green Master Mix.

SYBR Green RT-PCR:			
Gene Name	Gene Accession	Sense Primer	Antisense Primer
***β-Actin***	NM_031144.3	5′-CCC GCG AGT ACA ACC TTC T-3′	5′-CGT CAT CCA TGG CGA ACT-3′
***Acta2***	NM_031004.2	5′-GCC GAG ATC TCA CCG ACT AC-3′	5′-GTC CAG AGC GAC ATA GCA CA-3′
***Alex3***	NM_001014273.1	5′-GGT GAA GGT CGA GTT GAG GG-3′	5′-ACT TAT GCC ACA CCC AGC AA-3′
***CD206***	XM_032885181.1	5′-ACT GCG TGG TGA TGA AAG G-3′	5′-TAA CCC AGT GGT TGC TCA CA-3′
***Col1a1***	NM_053304.1	5′-TCC TGG CAA GAA CGG AGA T-3′	5′-CAG GAG GTC CAC GCT CAC-3′
***Crp***	NM_017096.3	5′-TGT CTC TAT GCC CAC GCT GAT G-3′	5′-GGC CCA CCT ACT GCA ATA CTA AAC-3′
***F4/80***	NM_001007557.1	5′-CTC TTC CTG ATG GTG AGA AAC C-3′	5′-CCC ATG GAT GTA CAG TAG CAG A-3′
***Fgf21***	NM_130752.1	5′-CAA ATC CTG GGT GTC AAA G-3′	5′-AAA GTG AGG CGA TCC ATA G-3′
***Gstm2***	NM_177426.1	5′-CCA AAC CTG AAG GAC TTC GT-3′	5′-GCC GCT CTT CAT GTA GTC AGA T-3′
***Il-1β***	NM_031512.2	5′-TGT GAT GAA AGA CGG CAC AC-3′	5′-CTT CTT CTT TGG GTA TTG TTT GG-3′
***MyD88***	NM_198130.1	5′-CCG TGA GGA TAT ACT GTA TGA ACT G-3′	5′-TTT CTG CTG GTT GCG TAT GT-3′
***Nox4***	NM_053524.1	5′-GAA CCC AAG TTC CAA GCT CA-3′	5′-GCA CAA AGG TCC AGA AAT CC-3′
***P22phox***	NM_024160.1	5′-GCC ATT GCC AGT GTG ATC TA-3′	5′-CTC AAT GGG AGT CCA CTG CT-3′
***Sirt1***	NM_001372090.1	5′-GAT ACC TTG GAG CAG GTT GC-3′	5′-CAC CTA GGA CAC CGA GGA AC-3′
***Birc5***	XM_032914019.1	5′-CTG GAC TGC CTT GAG GTG TA-3′	5′-TGAG TCC ATC GGC CTA GC-3′
***Tert***	NM_053423.1	5′-GGA TGT ACT TTG TTA AGG CAG-3′	5′-ATA TTG GCG ACA ATT TCC AC-3′
***Tgfβ 1***	NM_021578.2	5′-CCT GGA AAG GGC TCA ACA C-3′	5′-TGC CGT ACA CAG CAG TTC TT-3′
***Timp1***	NM_053819.1	5′-CAG CAA AAG GCC TTC GTA AA-3′	5′-TGG CTG AAC AGG GAA ACA CT-3′
***Tlr-2***	NM_198769.2	5′-ATG GGC TGT GGT ATC TGA GAA-3′	5′-AAA CAA AGG CAT CAT AGC AAA GG-3′
***Tlr-4***	NM_019178.1	5′-CGG AAA GTT ATT GTG GTG GTG T-3′	5′-GGA CAA TGA AGA TGA TGC CAG A-3′
***Tp53***	NM_030989.3	5′-ACA GCG TGG TGG TAC CGT AT-3′	5′-GGA GCT GTT GCA CAT GTA CT-3′

Acta2, Actin alpha 2 smooth muscle; Alex3, ARM protein lost in epithelial cancers on chromosome X 3; Birc5, survivin; CD206, macrophage mannose receptor 1; Col1α1, collagen type I alpha 1 chain; Crp, C-reactive protein; F4/80, adhesion G protein-coupled receptor E1; Fgf21, fibroblast growth factor 21, Gstm2, glutathione s-transferase mu2; Il-1β, interleukin 1 beta; MyD88, myeloid differentiation factor 88; Nox4, NADPH oxidase 4; P22phox, cytochrome B-245 alpha chain; Sirt1, sirtuin 1Tert, telomerase reverse transcriptase; Tgf β1, transforming growth factor beta 1; Timp1, TIMP metallopeptidase inhibitor 1; Tlr-2, toll-like receptor 2; Tlr-4, toll-like receptor 4; Tp53, tumor protein P53.

**Table 2:** Histopathologic characteristics of the livers of the rats fed with the experimental diets for 8 weeks.

	CC	HFHF	PT15	PT30	RSV30
**Lobular inflammation**					
None (0)	10	0	0	2	0
Mild (1)	0	5	8	8	7
Moderate (2)	0	5	2	0	3
**Ballooning degeneration**					
None (0)	10	0	0	0	0
Few (1)	0	0	0	0	0
Many (2)	0	10	10	10	10
**Fibrosis**					
Stage 0	0	9	10	10	10
Stage 1A	0	1	0	0	0
**NAS**	0 ± 0	5.4 ± 0.4	3.8 ± 0.3	3.1 ± 0.3	3.8 ± 0.3

Lobular inflammation was graded according to the number of inflammatory foci per 200 field (0 is no foci; 1 is < 2 foci per 200 × field; 2 is 2–4 foci per 200 × field); hepatocellular ballooning was graded as none (0), few balloon cells (1), and many cells/prominent ballooning (2); fibrosis stage was graded as none (Stage 0) or mild perisinusoidal fibrosis (Stage 1A); NAS: non-alcoholic fatty liver disease score.
